# Supplementary material for: Rapid Depletions of Subcutaneous Fat Mass and Skeletal Muscle Mass Predict Worse Survival in Patients with Hepatocellular Carcinoma Treated with Sorafenib
Source: Cancers (Basel). 2019 Aug 19;11(8):1206. doi: 10.3390/cancers11081206 (PMC6721466; doi:10.3390/cancers11081206)
Supplement: Supplementary file 1 [file cancers-11-01206-s001.pdf]

Figure S1. Kaplan-Meier curves for overall survival time for male.

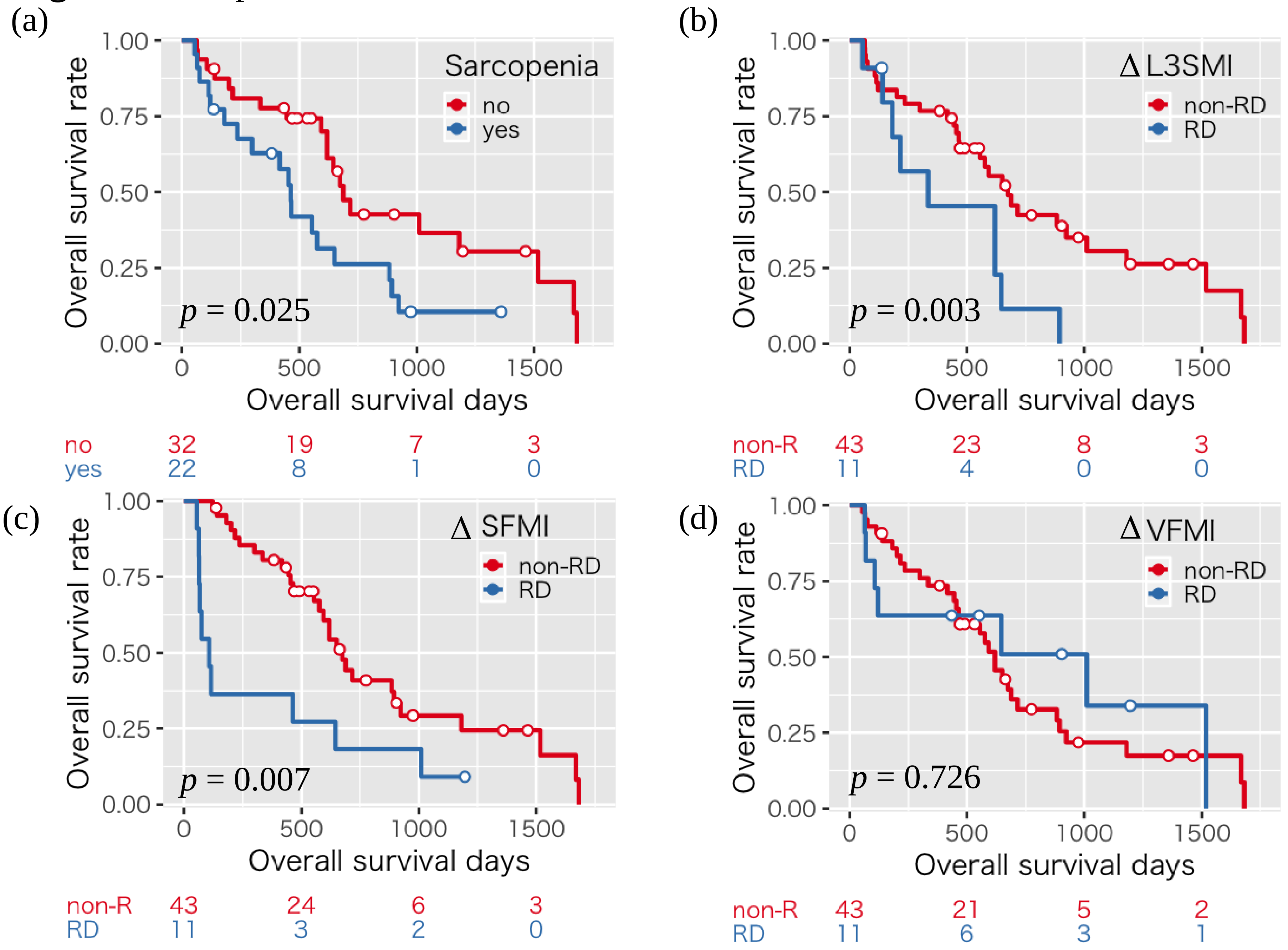

Figure S2. Kaplan-Meier curves for overall survival time for female.

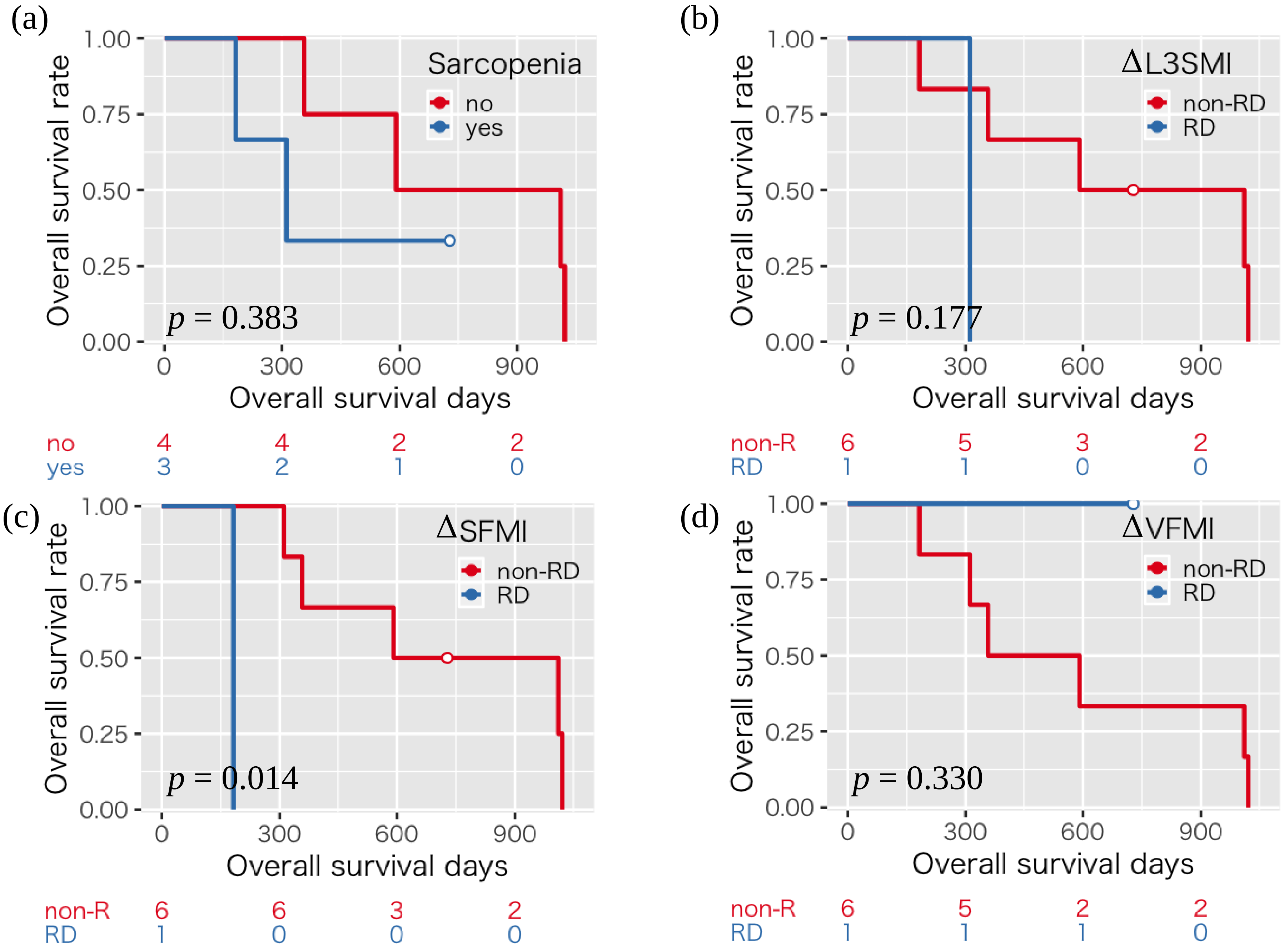

Figure S3. Intra-observer validations.

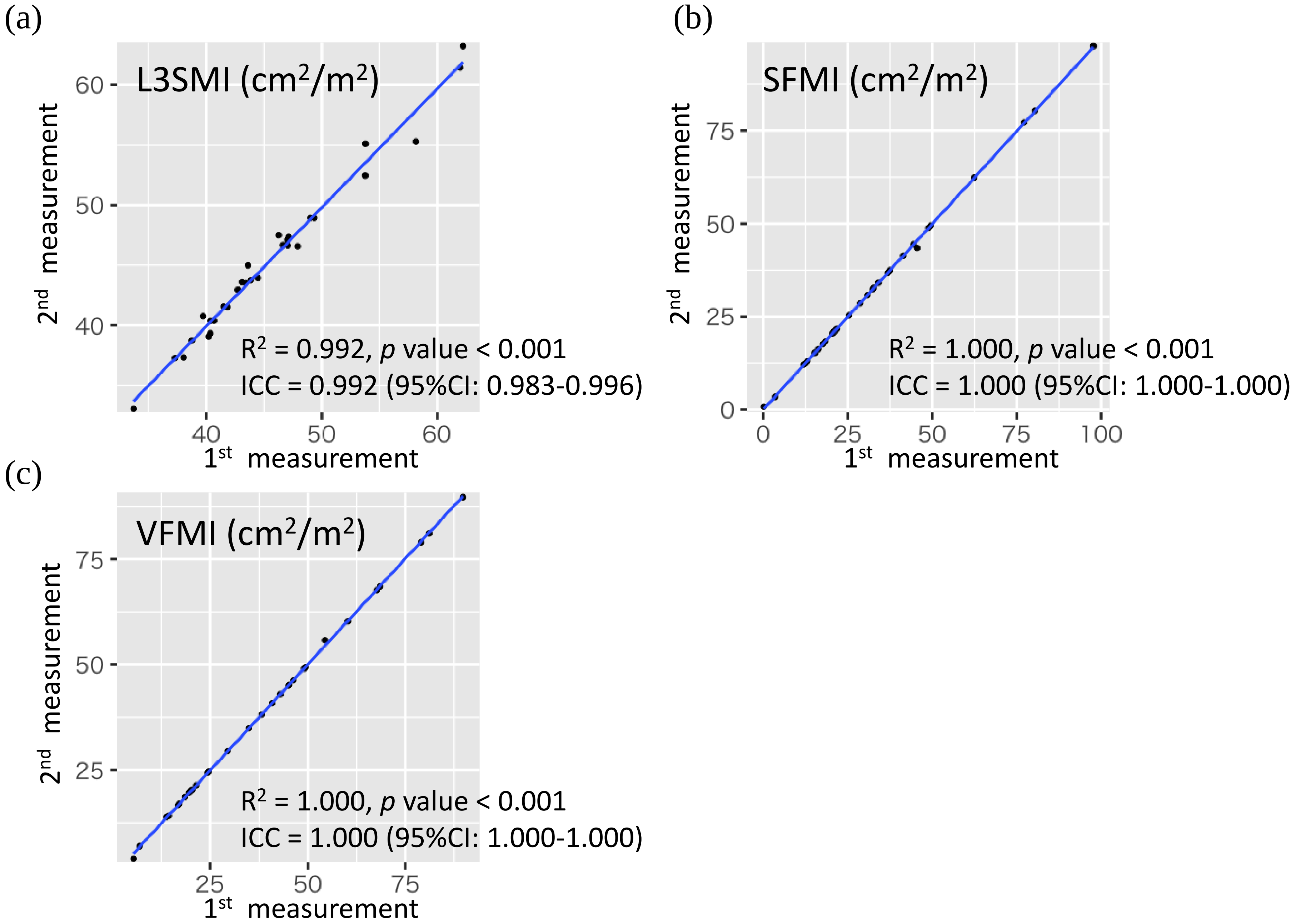

Table S1. The prevalence of patients with RD of ΔL3SMI, ΔSFMI, and ΔVFMI between sarcopenia and non-sarcopenia groups.

Variables	Non-Sarcopenia (n=36)	Sarcopenia (n=25)	*p* value
RD group in ΔL3SMI	7/36	5/25	>0.999
RD group in ΔSFMI	6/36	6/25	0.526
RD group in ΔL3VFMI	10/36	2/25	0.099
